# When PIP_2_ Meets p53: Nuclear Phosphoinositide Signaling in the DNA Damage Response

**DOI:** 10.3389/fcell.2022.903994

**Published:** 2022-05-13

**Authors:** Yu-Hsiu Wang, Michael P. Sheetz

**Affiliations:** Biochemistry and Molecular Biology Dept., University of Texas Medical Branch, Galveston, TX, United States

**Keywords:** p53, DNA repair, DNA damage response (DDR), nuclear phosphoinositide signaling, repair pathway choice, stress response

## Abstract

The mechanisms that maintain genome stability are critical for preventing tumor progression. In the past decades, many strategies were developed for cancer treatment to disrupt the DNA repair machinery or alter repair pathway selection. Evidence indicates that alterations in nuclear phosphoinositide lipids occur rapidly in response to genotoxic stresses. This implies that nuclear phosphoinositides are an upstream element involved in DNA damage signaling. Phosphoinositides constitute a new signaling interface for DNA repair pathway selection and hence a new opportunity for developing cancer treatment strategies. However, our understanding of the underlying mechanisms by which nuclear phosphoinositides regulate DNA damage repair, and particularly the dynamics of those processes, is rather limited. This is partly because there are a limited number of techniques that can monitor changes in the location and/or abundance of nuclear phosphoinositide lipids in real time and in live cells. This review summarizes our current knowledge regarding the roles of nuclear phosphoinositides in DNA damage response with an emphasis on the dynamics of these processes. Based upon recent findings, there is a novel model for p53’s role with nuclear phosphoinositides in DNA damage response that provides new targets for synthetic lethality of tumors.

## Introduction

### Nuclear Localization of Phosphoinositides and Their Metabolism

The existence of phospholipids in the nucleus was demonstrated in 1965 and 1977 (Rose and Frenster, 1965; Manzoli et al., 1977). A significant change in our understanding of nuclear phosphoinositides occurred in 1983 when Smith and Wells showed early evidence that the phosphoinositide kinases that generate Phosphatidylinositol (4,5)-bisphosphate were present in highly purified nuclear envelopes (Smith and Wells, 1983; Smith and Wells, 1984). ^32^P-Labelling of phosphatidic acid (PA), PI(4)P and PI(4,5)P_2_ occurred when intact rat liver nuclei were isolated and incubated with [γ^32^P]-ATP. It indicated that phosphoinositides can be synthesized in the nucleus in the absence of cytosolic phosphoinositide kinases. Later, adventurous studies by Irvine and coworkers lead to the discovery of the generation of phosphoinositides in the nucleus (Cocco et al., 1987; Divecha et al., 1991). The discovery of nuclear phospholipid signaling has revolutionized our view of the processes regulated by phospholipids. Interestingly, a substantial pool of nuclear phosphoinositides does not appear to be associated with the nuclear membrane (Payrastre et al., 1992). These observations are helping to unravel another facet of phospholipid signaling in cells.

To date, all the polyphosphoinositides, except PI(3,5)P_2_, have been detected in the nucleus using various approaches (Jacobsen et al., 2019). Further evidence that nuclear phosphoinositides constitute a different pool than those in the cytoplasm comes from the acyl compositions of nuclear PI(4,5)P_2_ (34:1) and (36:1), which is different from that of PI(4,5)P_2_ in the plasma membrane (38:4) (Ogiso et al., 2010). This finding further strengthens the hypothesis that nuclear phosphoinositides are generated through routes different from those in the plasma membrane.

Intracellular phosphoinositide localization in human cells has been investigated through imaging approaches using purified pleckstrin homology (PH) domains and antibodies (Boronenkov et al., 1998; Osborne et al., 2001; Kalasova et al., 2016). While phosphoinositides are not subject to formaldehyde fixation, an earlier study investigated the subcellular phosphoinositide distribution using ultrathin cryo-sections (Watt et al., 2002). The labelling procedure was carried out close to 0°C which overcame the issue that phospholipids could not be fully immobilized through aldehyde fixation. While the highest proportion of labelling of PI(4,5)P_2_ was over the plasma membrane (≈40% of total), the labelling in the nucleus was substantial (17–21% of total) and was particularly concentrated on electron-dense patches of heterochromatin (Watt et al., 2002). There is a tendency for immunolabelling to under-report the amounts of PI(4,5)P_2_ since it likely detects only freely exposed PI(4,5)P_2_ and not necessarily PI(4,5)P_2_ already bound to proteins. These and other findings from cellular fractionation and radioisotope labeling do not answer how nuclear phosphoinositides regulate nuclear function and in what form they exist in the nucleus (Divecha et al., 1993; Irvine, 2003; Barlow et al., 2010).

The nuclear phosphoinositide level is not kept constant. Accumulating evidence suggests that it fluctuates throughout the cell cycle (Clarke et al., 2001), plus levels respond to growth factor treatment (Divecha et al., 1991; Martelli et al., 1991; Martelli et al., 2000), cellular stressors (Jones et al., 2006), DNA damage (Jones et al., 2006; Li et al., 2012; Wickramasinghe et al., 2013; Wang et al., 2017; Choi et al., 2019), and differentiation (Cocco et al., 1987; Divecha et al., 1995; Neri et al., 2002). *In vivo* changes in phosphoinositides are also observed in liver nuclei in response to partial hepatectomy (Banfić et al., 1993; Neri et al., 1997). In this review, we will focus on the roles of phosphoinositides in the DNA damage response and summarize their responses at multiple time scales.

### Interactome Studies and General Functions

In an analysis of phosphoinositide-interacting proteins, the largest portion of these proteins identified were concentrated in the nucleus (Jungmichel et al., 2014). In total, 405 proteins were identified through a whole-cell stable isotope labeling with amino acids in cell culture (SILAC) proteome analysis that interacted with either PI(4)P, PI(4,5)P_2_ or PI(3,4,5)P_3_. A significant enrichment (*p* < 7.0 × 10^−7^) of phosphoinositide interactors was found in the nucleus in comparison to the total interactors among intracellular proteins (Jungmichel et al., 2014). Nearly 40% of them were either located in the nucleus or shuttled between nucleus and cytosol. In contrast, the density of cytosolic interactors was significantly lower compared to the intracellular distribution of overall proteins (*p* < 5.5 × 10^−3^). Lewis et al. characterized nuclear PI(4,5)P_2_ interactome and revealed that 168 out of 349 nuclear PI(4,5)P_2_-binding proteins isolated by neomycin extraction contained at least one phosphoinositide-binding domain, including plant homeodomain (PHD), PH or K/R-rich motifs (Lewis et al., 2011). It should be noted that enrichment of small nuclear ribonucleoproteins (snRNPs) are frequently found in such interactome studies and they typically colocalize with PI(4,5)P_2_ at nuclear speckles. A recent study from the same author(s) characterized the nuclear PI(3,4,5)P_3_ interactome that was composed of 179 proteins and identified new interaction partners associated with the nucleolus. A few DNA damage repair proteins including PARP1 were also identified (Mazloumi Gavgani et al., 2021). In an independent study, synthetic probes featuring PI(3,4,5)P_3_ headgroups with photoaffinity tags were used for covalently labeling PI(3,4,5)P_3_-binding proteins. An additional alkyne group in the probes allowed for conjugation with biotin that was used for further protein purification. This study revealed 265 PI(3,4,5)P_3_-interacting proteins. (Rowland et al., 2011). However, these two PI(3,4,5)P_3_ interactome studies shared few consensus proteins. Such a discrepancy could partially be explained by the difference in their approaches. In the work by Rowland et al., the synthetic probes were fed to the cells and UV crosslinked to capture any transient binding. This approach did not rely on protein-membrane interactions and could capture isolated protein-lipid complexes. In contrast, Gavgani et al. used phosphoinositide-coated beads for protein enrichment. The phosphoinositide lipids were in a membranous form, and might not have been able to capture freely diffusing protein-lipid complexes because of the lipid configuration. Another possible explanation is, however, the bifunctionally modified lipids failed to mimic natural phosphoinositides in their interactions with nuclear proteins. Although consensus on the nuclear phosphoinositide interactome has remained elusive, recent findings indicate that many phosphoinositide interactors largely reside in the nucleus.

Nuclear phosphoinositides potentially regulate nuclear functions by altering protein-protein or protein-DNA interactions (Viiri et al., 2012). Compared to the known physiological functions of phosphoinositides in the plasma membrane, very little is known about the role of phosphoinositide-protein interactions in the nucleus. Nuclear PI(4,5)P_2_ is central to nuclear phosphoinositide signaling not only because it is a second messenger but also because it interacts with a broad spectrum of nuclear proteins. The vast majority of nuclear PI(4,5)P_2_-interacting proteins contained lysine/arginine-rich patches with the following motif, K/R-(X_n=3–7_)-K-X-K/R-K/R, while a smaller subset of proteins contain known phosphoinositide-binding modules such as PH or PHD modules (Lewis et al., 2011). An analysis of over-represented biological processes for the PI(4,5)P_2_-interacting proteins, compared to all proteins annotated to the nucleus compartment, points to roles of nuclear PI(4,5)P_2_-interacting proteins in mRNA transcription regulation, mRNA splicing and protein folding (Lewis et al., 2011). So far, nuclear phosphoinositides have been shown to regulate many nuclear functions including transcription (Yu et al., 1998; Ulicna et al., 2018), splicing (Boronenkov et al., 1998; Osborne et al., 2001), export (York et al., 1999; Wickramasinghe et al., 2013) of mRNAs, as well as responses to genotoxic stress (Jones et al., 2013; Wang et al., 2017; Choi et al., 2019). Many facets of nuclear phosphoinositide functions have been widely reviewed (Fiume et al., 2012; Shah et al., 2013; Castano et al., 2019; Fiume et al., 2019; Jacobsen et al., 2019; Chen et al., 2020). In the following section, we will go through a brief summary of phosphoinositide-protein interactions and their functions in nucleus.

Nuclear PI(4,5)P_2_ appears to be involved in several important nuclear functions. First, nuclear phosphoinositides may regulate nuclear protein functions by altering protein-chromatin interactions (reviewed elsewhere (Hamann and Blind, 2018)). For example, PI(4,5)P_2_ interacts with histone H1 and disrupts its ability to suppress basal transcription by RNA polymerase *in vitro* (Yu et al., 1998). The interaction of PI(4,5)P_2_ with histone H1 is abolished upon Protein Kinase C (PKC)-mediated phosphorylation of H1. In addition, PI(4,5)P_2_ promotes chromatin association of the SWI/SNF-like chromatin remodeling complex BAF (Brahma-related gene association factor) upon lymphocyte activation (Zhao et al., 1998). BAF complex contains β-actin, BAF53 (an actin-related protein), and the ATPase subunit, BRG1. PI(4,5)P_2_ binding to the purified BAF complex subunit BRG1 stabilizes the complex by increasing its interaction with nuclear matrix and allows the complex to bind actin filaments (Rando et al., 2002). PI(4,5)P_2_ also interacts directly with the DNA Topoisomerase IIα (TopoIIα) and modulates TopoIIα decatenation activity *in vitro* (Lewis et al., 2011). Examples of nuclear PI(4,5)P_2_ mediated protein interaction with DNA are also found with the repression of transcription by WT1–BASP1, which requires the myristoylation of BASP1 and the PI(4,5)P_2_-dependent recruitment of HDAC1 (Toska et al., 2014). PI(4,5)P_2_ appears to be critical for forming protein-protein contacts between BASP1 and HDAC1. Both histone deacetylases (Gozani et al., 2003; Watson et al., 2012; Millard et al., 2013; Watson et al., 2016) and ATP-dependent chromatin remodeling activities (Zhao et al., 1998; Shen et al., 2003; Steger et al., 2003) require phosphoinositides or inositol phosphates for their activities.

In the case of PI(5)P, its binding to the PHD domain in ING2 protein promotes its association with chromatin (Gozani et al., 2003), whereas it inhibits chromatin-binding of another PHD-containing protein, ATX1 (Alvarez-Venegas et al., 2006; Ndamukong et al., 2010). PI(5)P also displaces the DNA from the zinc finger structure in SAP30 family members (Viiri et al., 2009). As an example, PI(3,4,5)P_3_ binds to the helix-turn-helix (HTH) structures in the homeobox (HOX) domains of HOXA5, HOXB6, HOXC6, and HOXD4. The competitive binding of phosphoinositides with target DNA sequences potentially modulates the transcription activities of these proteins (Bidlingmaier et al., 2011). They support the idea that the regulation of many nuclear proteins is mediated by their binding to phosphoinositides. Later, Divecha and Fischle et al. report two examples showing how PI(5)P could potentially regulate nuclear functions. In the first example, UHRF1 is shown to be allosterically regulated by PI(5)P for its interaction with unmodified histone H3 versus H3K9me3 by controlling their access to the PHD and tandem tudor domains (TTD), respectively (Gelato et al., 2014). In the second example, the basal transcriptional complex protein TAF3 is shown to directly bind to PI(5)P and transduce changes in nuclear phosphoinositides into differential transcriptional output that affects myoblast differentiation. The lipid kinase PIPKIIβ, phosphoinositides, and TAF3 form a conserved nuclear signaling pathway that selectively regulates transcription (Stijf-Bultsma et al., 2015).

Particularly interesting are the roles that nuclear phosphoinositides play in RNA function. PI(4,5)P_2_ interacts with the mRNA export protein, ALY, and thereby regulates selective mRNA export (Wickramasinghe et al., 2013). It also regulates RNA splicing and polyadenylation by interacting with RNA splicing complexes (Boronenkov et al., 1998; Bidlingmaier et al., 2011) and a non-canonical poly(A) polymerase, Star-PAP, also known as Tut1 (Mellman et al., 2008). Star-PAP modulates 3′-mRNA maturation and is strongly activated by nuclear PI(4,5)P_2_ (Mellman et al., 2008; Li et al., 2013; Li et al., 2017). Star-PAP interacts with PIPKIα through its C-terminus and colocalizes with PIPKIα at nuclear speckles (Mellman et al., 2008). *In vitro*, Star-PAP activity is dramatically stimulated by PI(4,5)P_2_, indicating that it is also a downstream target for nuclear PI(4,5)P_2_ signaling. These data indicate that the Star-PAP complex acts as a hub for nuclear PI(4,5)P_2_ signaling to control the response to oxidative stress by regulating the expression of PTEN (Li et al., 2017) and BIK, which is an important switch in the mitochondrial apoptosis pathway (Li et al., 2012).

### Connections Between Nuclear Phosphoinositides and DNA Damage Responses

The first connection that linked nuclear phosphoinositides to DNA damage response came from changes in the level of nuclear phosphoinositides following DNA damage induction. An early study reported increased synthesis of nuclear phosphoinositides after DNA damage. They used [γ^32^P]-ATP incorporation into PI(4,5)P_2_ that was detected using thin layer chromotagraphy (TLC) of isolated nuclei and found that nuclear PI(4,5)P_2_ level doubled within the first hour after damage by ionizing radiation (IR), but slowly returned to normal 18 h later in murine erythroleukemia cells (Rana et al., 1994). It was also noted that the nuclear PI(4,5)P_2_ accumulation preceded the marked increase in DNA synthesis after irradiation. This finding indicated that there is involvement of nuclear inositol lipids in the cascade of the early events leading to the regulation of DNA repair in the nucleus. (Rana et al., 1994). The mass of nuclear PI(5)P also increased two to four fold within 30 min following increased genotoxic stress induced either by UVC (254 nm), H_2_O_2_ or etoposide, a topoisomerase inhibitor, as determined by a radioactive mass assay. Interestingly, nuclear PI(5)P level remained unchanged after being exposed to γ-irradiation (from a Caesium-137 source) for up to 30 min (Jones et al., 2006). Our work showed that nuclear PI(4,5)P_2_ levels increased by about 80% within minutes and lasted for an hour following UVC irradiation, which preceded the increase of γH2AX, as revealed by immunofluorescence staining (Wang et al., 2017). This finding was in accordance with an earlier finding that tert-Butylhydroquinone (tBHQ), an oxidative stress inducer that also caused DNA damage, specifically promoted the generation of PtdIns(4,5)P_2_ in the nuclear speckles (Mellman et al., 2008). More recently, the same group also reported an increase in the nuclear PI(4,5)P_2_ level through type I PI4P-5K activation with cisplatin-induced DNA damage 24 h after treatment (Choi et al., 2019). Together, these observations strengthened a potential role of nuclear phosphoinositides in response to genotoxic and oxidative stress. However, it was also noted that H_2_O_2_ treatment triggered a 5-fold increase of PI(5)P at a whole cell level, which was accompanied by a global increase of PI(4)P by 70% (Chen et al., 2009; Jones et al., 2013) and a global decrease of PI(4,5)P_2_ by 35–50% (Halstead et al., 2006; Chen et al., 2009). The decrease of overall PI(4,5)P_2_ was possibly due to spleen tyrosine kinase (Syk)-mediated phosphorylation and deactivation of PIP5Kβ (Chen et al., 2009). It was not clear how nuclear PI(4,5)P_2_ levels varied in these studies. However, H_2_O_2_ likely triggered a broader oxidative stress response that included but was not limited to DNA damage. Whether nuclear PtdIns(4,5)P_2_ levels respond differently to different DNA damage induction mechanisms remains to be investigated.

Another connection between nuclear phosphoinositide and DNA damage response came from proteomic interactome studies. A nuclear PI(4,5)P_2_ interactome study was carried out using SILAC. In this proteome analysis, neomycin was used to release PI(4,5)P_2_-binding proteins in the nuclear fraction that were then pulled down using PI(4,5)P_2_-coated beads (Lewis et al., 2011; Rowland et al., 2011). This approach led to the identification of 168 nuclear proteins harboring lysine/arginine-rich patches or phosphoinositide-binding domains. Similar studies were performed recently using PI(3,4,5)P_3_-coated beads (Mazloumi Gavgani et al., 2021). This led to an identification of a small set of DNA damage repair proteins; including proteins from various repair pathways. For example, the non-homologous end-joining (NHEJ) proteins XRCC5 (Ku80), DNA-PKcs (PRKDC), NBN (NBS1), and potentially RECQL were present. Further, this included the homologous recombination (HR) proteins NBN and potentially RECQL, the single strand break repair (SSBR) proteins PARP1 and LIG3, the base excision repair (BER) protein MPG, and the translesion synthesis (TLS) protein RECQL. More generally, nuclear PI(3,4,5)P_3_ might have broadly impacted DNA repair processes, even if it was not directly required for the repair. The discovery of PARP1 as a PI(3,4,5)P_3_-interacting protein was exciting, since PARP1 was considered as the first responder to DNA damage. It mediated poly-ADP ribosylation (PARylation) of DNA repair proteins which promoted the recruitment of many repair proteins to DNA damage sites. PARP-mediated PARylation also mediated the rapid recruitment of p53 to DNA damage sites, which directly impacted repair pathway selection (Wang et al., 2022). A proteomic study that used anti-PI(4)P antibody for immunoprecipitation also led to the discovery of PI(4)P-interacting DNA repair proteins, which included homologous recombination protein, RPA3, and the translesion synthesis proteins, MCM4 and PCNA (Fáberová et al., 2020). Interestingly, immunostaining demonstrated different nuclear localizations of PI(4,5)P_2_ and PI(4)P during interphase and mitosis. Additionally, RPA3 was found to only interact with PI(4)P but not PI(4,5)P_2_. This indicated that there were functional differences between the two in the nucleus (Fáberová et al., 2020).

**FIGURE 1:**
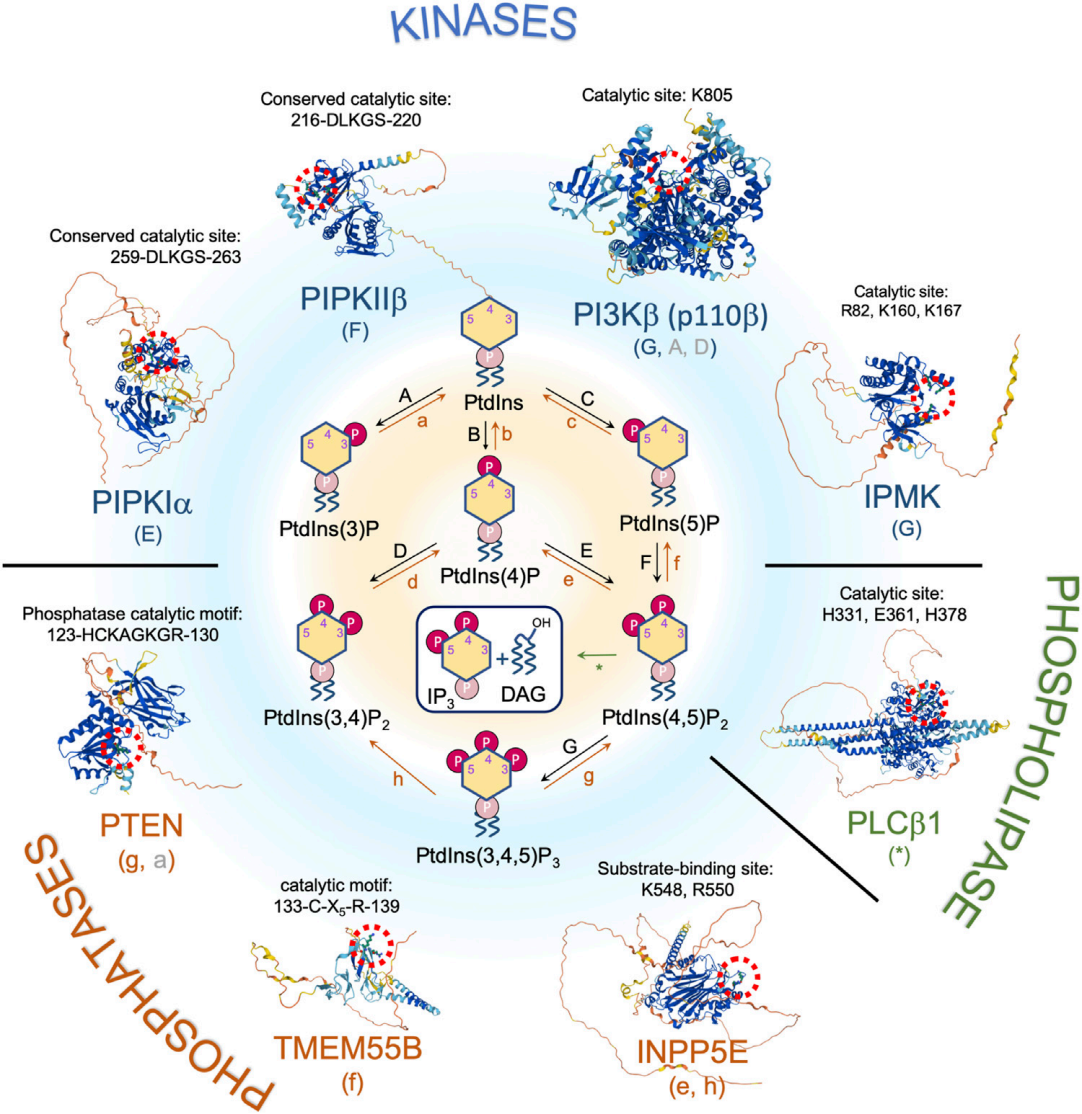
Nuclear phosphoinositide metabolism and involved enzymes with implications in DNA damage response and/or tumor suppression. Representative proteins/isoforms are shown as full length structures predicted by AlphaFold. Studies indicate that PIPKIα, PIPKIIβ, and PTEN might exist as dimers. Whether dimerization is required for their kinase activities and in response to DNA damage requires further investigation (Heinrich et al., 2015; Hu et al., 2015; Droubi et al., 2016). Red dotted circle labels the catalytic site of each protein. The letters in the parentheses under each enzyme correspond to the pathways indicated in the center panel. Letters in gray represent a minor pathway. Note that PI(3,5)P_2_ is not detected in nucleus.

Further investigation of cells under genotoxic conditions might lead to discovery of more PI(3,4,5)P_3_-interacting proteins that are involved in the DNA damage repair, since many repair proteins go through post-translational modification following damage induction. A study identifies DNA polymerase kappa (Polκ/POLS) as a phosphoinositide-binding protein by using a yeast surface displayed cDNA library exon microarray to probe for phosphoinositide-protein interactions (Bidlingmaier et al., 2011). Polκ is a Y-family DNA polymerase involved in TLS, which bypasses DNA lesions during replication. It is therefore possible that nuclear phosphoinositides might play a role in TLS. Nuclear lipids potentially also regulate nuclear functions by inducing intranuclear phase separations, which is reviewed elsewhere (Sztacho et al., 2019). Whether nuclear phosphoinositides actually regulate pathways that involve the above-mentioned DNA repair proteins requires further investigation. A schematic summary of nuclear phosphoinositide metabolism along with several key nuclear phosphoinositide-metabolizing enzymes that respond to DNA damage is shown in Figure 1. How these enzymes are regulated in response to DNA damage will be discussed in later sections.

### Methodology

The general methodology for studying nuclear lipids is nicely summarized in a recent review (Moriel-Carretero, 2021). Here we briefly go through common techniques that are available for studying the roles of nuclear lipids in DNA damage repair. The methods can be put into two categories; the first measures the level changes and/or intranuclear localization of the nuclear phosphoinositides and the second involves antibody-based techniques. The methods in the first category are well developed. Radioisotope labeling using isolated nuclei detects newly phosphorylated phosphoinositides. To measure the mass level of individual nuclear phosphoinositide lipids requires nuclear isolation and lipid extraction using Bligh-Dyer or Folch methods followed by de-acylation before liquid chromatography analysis (Jones et al., 2013). However, this method can be strongly affected by the quality of samples, since residual ER membrane might remain attached to the nuclear envelope, which results in contamination from the cytosolic membranous organelles. Additionally, isolation procedures can result in the loss of nuclear phosphoinositides that form complexes with free-diffusing nuclear proteins such as nuclear receptors, NR5A1 (SF1) and NR5A2 (LRH1). (NR5A1 is also known as steroidogenic factor 1 (SF1), but SF1 now more commonly refers to splicing factor 1). In the second case of antibody-based methods including immunostaining, immunogold labeling for electron microscopy, and recently the popular proximity ligation assay (PLA), staining of phosphoinositides relies on the fixation of their binding proteins since lipids are not subject to formaldehyde fixation. Thus, staining might not reveal the genuine intranuclear distribution of phosphoinositides but rather gives indirect information.

Another set of methods involves the investigation of nuclear phosphoinositide-protein interactions. While PLA is also used for this purpose, the throughput is very limited. Instead, mass spec-based proteomic studies provide a more global picture of the phosphoinositide interactomes. In this case, neomycin extraction of nuclear fractions is used for phosphoinositide-binding protein enrichment. The sample is then further purified by affinity-based purification with phosphoinositide lipid-coated beads. Depending on the phosphoinositide on the beads, the binding specificities of nuclear proteins can be investigated. This approach is suitable for proteins that form strong and stable interactions with the phosphoinositide lipids in a bilayer form. For transient protein-lipid interactions, chemically modified bifunctional phosphoinositide lipids are used (Haberkant et al., 2013). Such lipids carry a photoactivable group that allows UV-induced crosslinking between the lipids and protein, and an alkyne group that allows for Click reaction for affinity column purification. These so called “clickable” lipids are commercially available. The main drawback of this system is that any chemical modification either at the head group or acyl chain of the phospholipids might lead to a dramatic change in their physical chemical properties, as the modifications are often comparable in size to a lipid head group.

Most methods do not allow us to probe for the early dynamics of nuclear phosphoinositides in the DNA damage response. Alternatively, genetically encoded phosphoinositide-sensing probes with fluorescent tags allow for visualizing phosphoinositides in living cells. This is made possible with the significant contributions from the Hammond and Balla laboratories [reviewed elsewhere (Hammond and Balla, 2015)]. Further, nuclear localization signal (NLS)-tagged phosphoinositide-binding domains fused with fluorescent proteins provide rapid sensing of lipid distributions in the nucleus. However, the major limitation comes from the high background noise of the lipid-free fluorescent proteins in the nucleoplasm. SV40-derived NLS-tag commonly leads to artefactual accumulation of target proteins at nucleoli (Kitamura et al., 2015). As a result, only significant changes in the localization of the phosphoinositides in the nuclear matrix become detectable (Kalasova et al., 2016; Wang et al., 2017). Much better results can be obtained with strong focused perturbations such as laser microirradiation to generate localized DNA damage. With that system, the changes in the local concentrations of the phosphoinositides and the recruitment of DNA repair proteins can be detected in live-cell imaging. The overexpressed lipid-binding domains also serve as sequestering agents. The expression of these probes mimics the depletion of nuclear phosphoinositides and allows for the investigation of the role of individual lipid species. While certain kinases and phosphatases such as PTEN can utilize both proteins and lipids as substrates (Bassi et al., 2013), this approach is advantageous because it helps to see the dual roles of such signaling proteins without perturbing their expression levels.

## Involvement of Nuclear Phosphoinositides in DNA Damage Signaling

In the next section, we will discuss studies using nuclear phosphoinositide binding domains to probe for roles of nuclear phosphoinositides in DNA damage signaling. The changes in the local enrichment of DNA repair protein provide clues about how phosphoinositides are involved at different stages of DNA damage response.

### Nuclear Phosphoinositide Sequestering Alters DNA Damage Signaling

**FIGURE 2:**
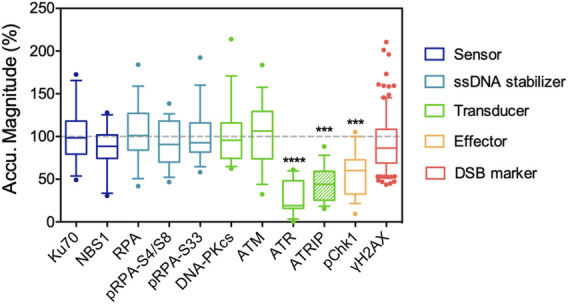
Nuclear PI(4,5)P_2_ sequestering by NLS-tagged PLCδPH domain suppresses the recruitment of ATR/ATRIP complex following UVA laser microirradiation, and the accumulation of its downstream effector, phospho-S345 Chk1. The recruitment of ATM, DNA-PKcs and other DNA repair proteins listed here are not affected. The data is adapted with permission from (Wang et al., 2017).

Accumulating evidence indicates that increases in nuclear phosphoinositides are part of the DNA damage response, or in a broader term, stress response. The elevation of nuclear phosphoinositide influences gene regulation in many studies, which leads to stabilization (Choi et al., 2019) and activation of p53 (Gozani et al., 2003) or the expression of the pro-apoptotic protein, Bcl2-interacting killer (BIK) (Li et al., 2012). The information regarding how phosphoinositides affect DNA damage repair per se is rather limited. Our previous work focused on how nuclear phosphoinositide sequestering affected repair protein recruitment. Instead of depleting relevant phosphoinositide kinases and phosphatases, we used NLS-tagged phosphoinositide-binding domains and laser microirradiation to interrogate the role of individual nuclear phosphoinositides in DNA damage repair (Wang et al., 2017). The key finding was that the sequestering of nuclear PI(4)P, PI(4,5)P_2_ or PI(3,4,5)P_3_ suppressed ATR recruitment to different degrees. Sequestration of nuclear PI(3,4,5)P_3_ was the most effective, followed by PI(4,5)P_2_ and then PI(4)P. Among the three major PI3K-like kinases: ATR, ATM and DNA-PKcs that served as DNA damage signal transducers, only the recruitment and activation of ATR was significantly suppressed by sequestration of phosphoinositides. The local accumulation of ATR together with its interacting protein, ATRIP, was suppressed by more than 80%. Activation of ATR, as indicated by the phosphorylation of Chk1, was also significantly suppressed (Figure 2). Since all experiments were performed 6–12 h post transfection, we expected that the cellular protein expression profile was not significantly altered by the NLS-tagged PH or P4M domains. Interestingly, the recruitment of ATR was typically mediated by the binding between ATRIP and RPA in a canonical pathway. However, in this study, the recruitment of RPA remained unaffected and only the recruitments of ATRIP and ATR were suppressed. This indicated that nuclear phosphoinositide sequestration directly affected protein-protein interactions in the DNA damage repair network.

### Nuclear Phosphoinositides Mediate DNA Damage Response at Multiple Time Scales

**FIGURE 3:**
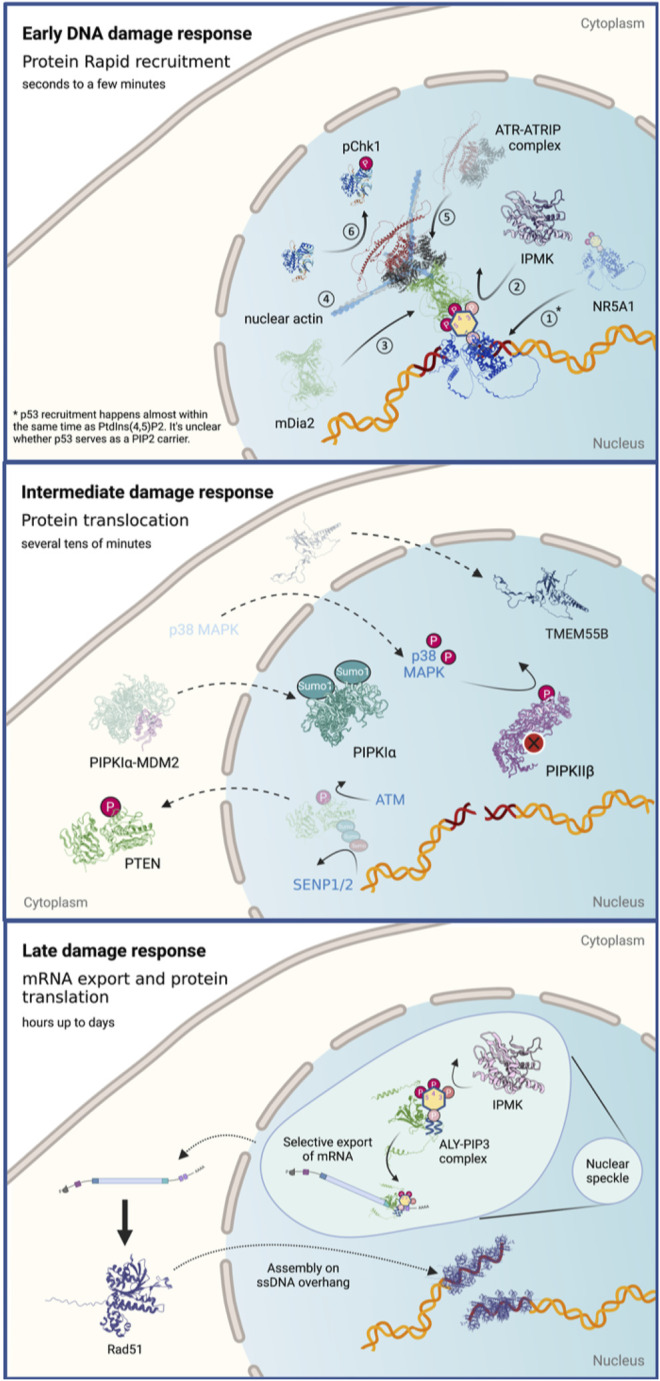
The roles of nuclear phosphoinositides and the regulation of their kinases and phosphatases in response to DNA damage at different time scales. Figures created with BioRender.com.

In this section, we would like to summarize the current findings regarding the roles of phosphoinositides in DNA damage at different time scales. Many studies have probed the roles of phosphoinositides in stress responses including DNA damage; however, those studies cover a wide range of time scales. To get a larger view of what happens with phosphoinositide levels and the relevant enzymes in the nucleus, we dissect this process into three stages that are discussed separately (Figure 3).

#### Early Damage Responses

We define the early DNA damage response as events that happen within the first 1–3 min following damage induction. The damage response at this stage mainly involves intranuclear redistribution and local accumulation of the nuclear factors at damage sites.

Our knowledge of the roles of nuclear phosphoinositides at this time scale is rather limited because there are few relevant assays. UVA laser microirradiation at 355 nm and localization of GFP-tagged phosphoinositide-binding domains document accumulation of phosphoinositides at damage sites. Phosphoinositide-binding domains rapidly accumulate at DNA damage sites within seconds and the accumulation of phosphoinositide drives the recruitment and activation of ATR. Both PI(4,5)P_2_-binding PLCδ-PH and PI(3,4,5)P_3_-binding Btk-PH domains are rapidly recruited to damage sites with similar dynamics. The magnitude of Btk-PH domain accumulation decreases after wortmannin inhibition or IPMK depletion, suggesting the involvement of PI3K activity in this process. Since IPMK doesn’t accumulate at damage sites, IPMK might help to maintain the PI(4,5)P_2_/PI(3,4,5)P_3_ ratio and nuclear phosphoinositide homeostasis. The phosphoinositide accumulation is driven, at least partially, by a nuclear receptor NR5A1, which is a known substrate of IPMK and a PI(4,5)P_2_/PI(3,4,5)P_3_ carrier through its acyl chain-binding domain. It remains unclear how phosphoinositides are loaded into NR5A1. Intriguingly, SUMOylation of NR5A1 drives NR5A1 to localize at nuclear speckles (Chen et al., 2004). Hence, NR5A1 SUMOylation provides a plausible mechanism by which NR5A1 can be loaded with PI(4,5)P_2_ *via* direct uptake or by the action of phospholipid transport proteins (PLTPs) at nuclear speckles (Davis et al., 2015). Whether inhibition of NR5A1 by SUMOylation alters DNA repair requires further investigation. However, it should be noted that the distribution of NR5A1 in human body is highly uneven. It is concentrated only in a handful of tissues which includes endocrinal tissues, bone marrow, lymphoid tissues and reproductive organs. Therefore, whether NR5A1-medaited early DNA damage signaling serves as a general mechanism in all tissues remains a question. While this study does not exclude the involvement of other phosphoinositide carriers, the involvement of other phosphoinositide carriers remains to be explored. Interestingly, the recruitment dynamics of phosphoinositides are almost identical to those of recruitment of p53. It is however not clear whether p53 serves as a PI(4,5)P_2_ carrier during the early DNA damage signaling events. Both RNAi depletions of IPMK or NR5A1 suppress ATR activation, which supports the proposed pathway.

To address how nuclear phosphoinositide accumulation drove ATR recruitment, we investigated the involvement of nuclear actin, whose assembly was induced by DNA damage and was critical for DNA damage clearance (Belin et al., 2015). Evidence of the involvement of nuclear actin assembly in ATR recruitment came from Latrunculin A (Lat A) inhibition. Actin disassembly by Lat A suppressed both ATR recruitment and Chk1 phosphorylation. While effects of Lat A on disrupted nuclear 3D architecture cannot be excluded, our observation is consistent with earlier findings that introduction of nuclear-targeted mutant actin that cannot polymerize, or the depolymerization of endogenous actin filaments by the addition of cytochalasin D (Cyto D) or Lat A, promotes chromatin association of DNA damage repair proteins from both the NHEJ and the HR pathways following ionizing irradiation. It also leads to prolonged retention of Ku80 at sites of damage in live cells (Andrin et al., 2012). Depletion of IPO9, a nuclear actin import factor, leads to delayed removal of 53BP1 and γH2AX foci (Belin et al., 2015). It’s also in line with the observation that the overexpression of cofilin, an actin-depolymerizing factor, alters nuclear actin dynamics and sensitize cells to radiation induced DNA damage (Lee et al., 2005). While the involvement of nuclear actin in the DNA damage response has drawn increasing attention in recent years, the identification of a nucleation polymerization factor (e.g., N-WASP, Formins or Arp2/3) remains controversial [see (Caridi et al., 2019) for a related review]. We found that nuclear phosphoinositide accumulation was followed by the accumulation of nuclear formins and nuclear actin, thereby completing the signaling axis (Wang et al., 2017).

The rapid local accumulation of PI(3,4,5)P_3_ at damage sites was also reported in an independent study. In a study by Kumar et al., class IA PI3Kβ (p110β) and PI(3,4,5)P_3_ both accumulated at DNA damage sites after either UV or IR irradiation (Kumar et al., 2010). Depletion of p110β lead to inhibition of ATM and ATR repair pathways as indicated by the suppressed phosphorylation of downstream effectors. p110β was the PI3K isoform that predominantly resided in the nucleus. Further investigation revealed that purified p110β interacted with Rad50 (an MRN complex component), Rad17 (an ATR effector), and Rad9B. Endogenous p110β interacted with both Rad50 and Nbs1 in intact nuclei upon UV or IR treatment. The recruitment of Nbs1 to damage sites following laser microirradiation depended on the expression, but to a lesser degree on the kinase activity of p110β. This indicated that p110β governed Nbs1 recruitment through a protein-protein interaction that was independent of its kinase activity. Finally, p110β deletion was shown to induce genomic instability, which strengthened its role in the DNA damage response. Twenty percent of untreated mouse embryonic fibroblasts (MEF) derived from p110β^−/−^ mice had spontaneous DNA lesions in a comet assay, but not MEF cells derived from WT or kinase-dead p110β mice. It was therefore concluded that p110β expression was required for a DDR process, in which its kinase activity was not required. These results indicated that class I PI3Kβ was a critical factor for genomic integrity.

Interestingly, the recruitment dynamics of p110β and nuclear PI(3,4,5)P_3_, as determined using a BtkPH-EGFP construct, were around 50 s and 25 s, respectively, after UVA laser microirradiation (365 nm) (Kumar et al., 2010). The fact that the halftime of p110β recruitment was longer than that of BtkPH indicated that other enzymes with PI3K activity such as IPMK were involved in this process. This supported the hypothesis that p110β′s role in DDR was essentially kinase-independent, although its kinase activity facilitated the repair process. Additionally, the recruitment dynamics of BtkPH, and therefore PI(3,4,5)P_3_, from this study were significantly slower than those in our study (Wang et al., 2017). It should be noted that comparing results from different laser microirradiation studies is usually very difficult due to the lack of standardized parameters (e.g., laser wavelength, pulse width, repetition rate, power delivered at the back focal plane of the objective etc.). Comparisons of recruitment dynamics of different DNA repair components are largely limited to results from the same system.

#### Intermediate Damage Responses

The intermediate DNA damage response is defined as the events that happen within tens of minutes of the damage. At this time scale, the changes in phosphoinositide metabolism, and therefore their levels in the nucleus, mainly involve the posttranslational modification and translocation of phosphoinositide kinases and phosphatases.

In the following section, we examine how the levels of individual phosphoinositide species change, followed by how its regulation is altered in response to DNA damage. Starting with PI(5)P, the level of nuclear PI(5)P increased by 4–6 fold within 20–40 min following H_2_O_2_ treatment. This increase correlated with a small but significant decrease in PI5P-4K activity through p38 MAPK-mediated phosphorylation of PIPKIIβ at Ser326 and Thr322 (Jones et al., 2013). The nuclear translocation of p38 MAPK was driven by DNA damage induced activation and conformational change after exposure to ionizing irradiation or UV (Wood et al., 2009). p38 MAPK was activated through phosphorylation at Thr180/Tyr182 by upstream MAPKKs (Raingeaud et al., 1995). The phosphorylation of PIPKIIβ by nuclear p38 suppressed PIPKIIβ kinase activity by about 40% (Jones et al., 2013). As the change in PIPKIIβ activity was relatively minor, the significant increase in PI(5)P levels likely also involved the nuclear import of TMEM55B, also known as PIP4P1, which is a type I PI(4,5)P_2_ 4-phosphatase. TMEM55B predominately localized in the cytosol of cells in a resting state (Ungewickell et al., 2005). The nuclear fraction of TMEM55B increased from 15 to 40% within 4 h in cells exposed to etoposide. The same phenomenon has been observed in both the endogenous and overexpression systems. Since the total amount of TMEM55B appeared unchanged, an increase in nuclear TMEM55B likely resulted from the redistribution of this enzyme (Zou et al., 2007). The resulting increase of PI(5)P created a positive feedback loop by driving p38-dependent activation of a nuclear ubiquitination ligase complex, SPOP-Cul3, which promoted the degradation of PIPKIIβ (Bunce et al., 2008). In summary, the nuclear PI(5)P increase in response to DNA damage was jointly regulated by the inactivation and down-regulation of PIPKIIβ as well as the nuclear import of TMEM55B.

It should be noted that the type of DNA damage also plays a role in the damage response dynamics. Ionizing radiation and UV share an advantage that there is clear time zero in the sequence of events. However, ionizing radiation produces various types of DNA damage, which include base damage, apurinic/apyrimidinic (AP) sites, DNA single-strand breaks (SSBs), DNA double-strand breaks (DSBs), and DNA–protein crosslinks (DPCs) (Nakano et al., 2022). The same complexity is also seen with UV-induced damage, but the types of damage strongly depend on the wavelength. UVA induces both UV-specific DNA adducts (such as cyclobutane pyrimidine dimers (CPDs) and pyrimidine-pyrimidone (6–4) photoproducts) and oxidative damage in a 1:1 ratio, while the latter includes a wide spectrum of DNA products (Cooke et al., 2003). UVC is 10,000 times more effective than UVA and 100 times more effective than UVB in DNA adduct formation. The DNA adduct:oxidative damage ratio reaches 100:1 in UVC-induced damage *in vitro* (Kuluncsics et al., 1999). Therefore, UVC is commonly used if UV-specific damage is to be studied. By contrast, DSBs induced by etoposide slowly increase and reach a maximal level after 40 min, but H2AX phosphorylation continues to increase and reaches its maximum only after 160 min (Muslimović et al., 2009). Additionally, etoposide causes both SSBs and DSBs with DSBs being a minor product (∼3%). Alternatively, several chemical agents are considered radiomimetic drugs in that they imitate the effect of ionizing radiation and break the DNA directly. Radiomimetic drugs include bleomycin, phleomycin and neocarzinostatin, which attack DNA directly and create DSBs within the first few minutes (Vítor et al., 2020). To better assess the dynamics of DSB repair, chemicals like neocarzinostatin are recommended, as its excess will be quickly hydrolyzed in the medium. Different types of damage effectors have different kinetics.

Secondly, we considered the regulation of PI(4,5)P_2_ at an intermediate time scale. While it was clear that nuclear PI(4,5)P_2_ played a role in the DNA damage response, how nuclear PI(4,5)P_2_ level was regulated upon DNA damage remained elusive. The level of nuclear PI(4,5)P_2_ increased by about 80% shortly after being exposed to UVC (within minutes) and colocalized with DNA damage sites (γH2AX foci) (Wang et al., 2017). The finding was later confirmed by a study in which elevation of nuclear PI(4,5)P_2_ was also observed in cells treated with cisplatin for 24 h. Additionally, PIPKIα, PI(4,5)P_2_, and p53 strongly colocalized with γH2AX foci upon cisplatin treatment (Choi et al., 2019). The dynamic aspect of this process has not been investigated in detail. Recent evidence indicated that the regulation of PIPKIα activity was mediated by complex formation and was context dependent. PIPKIα was activated in different complexes. Two scenarios of upregulated PIPKIα activity were described in response to DNA damage. One was mediated by complex formation with p53 and small heat shock proteins (sHSP) (Choi et al., 2019). DNA damage induced dissociation of PIPKIα from MDM2 in the cytosol and promoted nuclear translocation of PIPKIα. The association of PIPKIα and p53 in the nucleus mediated p53-PI(4,5)P_2_ complex formation, which promoted the recruitment of sHSP thereby stabilizing p53 following cisplatin treatment (Choi et al., 2019). How PI(4,5)P_2_ interacted with p53 will be further discussed in a later section. Another PIPKIα activation mechanism in response to DNA damage was reported by the same group in a different context. In this other scenario, DNA damage induced by etoposide treatment for 4 h triggered a nuclear translocation of PKCδ, which formed a complex with PIPKIα and Star-PAP. PI(4,5)P_2_ produced by PIPKIα activated PKCδ. Activated PKCδ in turn activated Star-PAP for the 3’ end processing of the mRNA (Li et al., 2012). This still begs the question of how PI(4,5)P_2_ level is regulated by damage.

Another aspect of nuclear PI(4,5)P_2_ signaling is the regulation of nuclear 5′-phosphatases, which include SHIP1&2 and INPP5E. SHIP1 is present in nucleoli (Ehm et al., 2015). Phosphorylated SHIP2 (pSer132) colocalizes with PI(4,5)P_2_ in nuclear speckles (Déléris et al., 2003), which implies PI(4,5)P_2_ phosphatase activity is present (Taylor et al., 2000; Elong Edimo et al., 2011). Conversely, a substrate analysis of SHIP2 using the recombinant SHIP2 catalytic domain *in vitro* showed the following preference: Ins(1, 2, 3, 4, 5)P5 > Ins(1, 3, 4, 5)P4 > PI(3,4,5)P_3_ ≈ PI(3,5)P_2_ ≈ Ins(1, 4, 5, 6)P4 > PI(4,5)P_2_ (non-detectable) (Chi et al., 2004). Therefore, whether SHIP2 is the major nuclear 5′-phosphatase remains debatable and whether it responds to genotoxic stress needs to be investigated. The type IV 5′-phosphatase, INPP5E, on the other hand, shows a stronger connection with DNA damage repair and preserving genome stability. It utilizes PI(3,4,5)P_3_ as its primary substrate, and has also been shown to dephosphorylate PI(4,5)P_2_ and PI(3,5)P_2_. INPP5E localizes to centrosomes, chromosomes, and kinetochores in early mitosis and shuttles to the midzone spindle at mitotic exit. It supports genomic stability through regulation of mitosis (Sierra Potchanant et al., 2017). Knockdown of INPP5E causes chromosomal instability in primary human fibroblasts. Micronucleus assays reveal increased frequencies of both mitotic errors and unrepaired double-strand DNA breaks in INPP5E-deficient cells (Sierra Potchanant et al., 2017). Additionally, INPP5E is phosphorylated at ATM/ATR recognition sites following exposure to ionizing radiation (Matsuoka et al., 2007), but how ATR-mediated phosphorylation regulates INPP5E activity is not yet understood in depth. Thus, the roles of the nuclear 5′-phosphatases in the DNA damage response are not well understood.

The last issue in the intermediate timescale is the regulation of PI(3,4,5)P_3_. In addition to the early recruitment to DNA damage sites of p110β, which coincides with local enrichment of PI(3,4,5)P_3_ (Kumar et al., 2010), the 3′-phosphatase PTEN also plays a role in mediating DNA damage response. The roles of PTEN in the DNA damage response involve both phosphatase-dependent and phosphatase-independent functions. Further, the PTEN story is complicated by the fact that PTEN possesses both lipid and protein phosphatase activities. Although it’s getting clear that nuclear PTEN is important for efficient DNA repair, chromosome stability, and cell cycle progression (reviewed elsewhere (Milella et al., 2015)), an increasing body of evidence suggests that nuclear PTEN mediates DNA damage response largely through a lipid phosphatase-independent activity (Bassi et al., 2013; Ma et al., 2019). Although PTEN has been shown to act on PI(3,4,5)P_3_-NR5A1 complexes in cell studies (Blind et al., 2012), an earlier report indicates that there is an independent pool of nuclear PI(3,4,5)P_3_ that is insensitive to PTEN (Lindsay et al., 2006). These findings argue that PTEN is more than a lipid phosphatase in the DNA damage response.

The phosphatase-independent functions of PTEN in the DNA damage response often involve post-translational modification and nuclear translocation of PTEN. Despite the absence of a classic nuclear localization signal, a few nuclear translocation mechanisms of PTEN have been proposed. These include simple diffusion, active shuttling, cytoplasmic localization signal-dependent export and post-translational modification-dependent import (Planchon et al., 2008). It has been shown that SUMOylation (SUMO, small ubiquitin-like modifier) of PTEN at Lys254 by UBC9 drives its nuclear localization in HEK293 cells (Bassi et al., 2013). Ionizing radiation causes a gradual reduction in the amounts of SUMO-PTEN, and therefore depletion of nuclear PTEN beginning 1 h after IR exposure with the steady-state amounts returning 8 h later. Other forms of genotoxic stress, such as treatment with cisplatin or doxorubicin, also cause depletion of SUMO-PTEN, in conjunction with the appearance of DNA damage elicited by these agents. ATM-mediated phosphorylation of PTEN at Thr398 promotes the deSUMOylation of PTEN, which is mediated by SENP1/2 (Bassi et al., 2013; Bawa-Khalfe et al., 2017), and therefore facilitates the nuclear export of PTEN in response to IR. Cells lacking nuclear PTEN are hypersensitive to DNA damage, whereas PTEN-deficient cells are susceptible to killing by a combination of genotoxic stress and a small-molecule PI3K inhibitor both *in vitro* and *in vivo* (Bassi et al., 2013). Importantly, a PTEN mutant lacking the lipid phosphatase activity demonstrates similar DNA damage clearance dynamics as the wildtype, while the mutant lacking both lipid and protein phosphatase activities demonstrates decreased HR efficiency, similar to the PTEN-depleted cells (Bassi et al., 2013). In another example, ionizing radiation leads to FGFR2-mediated tyrosine phosphorylation of nuclear PTEN at Y240, which happens independently of the SUMOylation of PTEN at K254 (Ma et al., 2019). Inhibition of Y240 phosphorylation using FGFR inhibitors sensitizes tumors to ionizing radiation. pY240-PTEN binds to chromatin through interaction with Ki-67 and promotes HR-mediated DSB repair by facilitating RAD51 filament formation, which requires neither protein nor lipid phosphatase activity of PTEN (Ma et al., 2019).

Some earlier studies that reveal the role of PTEN in DNA damage repair didn’t look into the dependence of its phosphatase activity and are summarized here. In addition to the role of PTEN in homologous recombination, PTEN also positively regulates global genome NER (GG-NER) by promoting XPC transcription in keratinocytes (Ming et al., 2011; Ming and He, 2012). Inhibition of PTEN impairs GG-NER capacity through suppressing the expression of XPC. The PTEN/AKT/p38 axis seems critical for regulating XPC levels and therefore GG-NER capacities (Ming et al., 2011). These studies demonstrate that cells deficient in nuclear PTEN have defective DNA repair, consistent with the genomic instability phenotype in PTEN-depleted cells (Shen et al., 2007). Altogether, although the roles of nuclear PTEN in DNA damage repair are largely phosphatase-independent, PTEN has clinical value in DNA damage-based cancer treatment through synthetic lethality with FGFR (which governs the phosphorylation of PTEN at Y240) and/or PI3K inhibitors (Bassi et al., 2013).

#### Late Damage Responses

We define the late damage responses as the events that happen from several hours to even days following damage induction. They include the two PIPKIα activation mechanisms mentioned in the previous section that are mediated by complex formation with PLCδ and p53, respectively. The former involves the nuclear import of PLCδ, which leads to the activation of Star-PAP and elevation of BIK mRNA and protein level within 4 h after etoposide treatment. The latter involves p53 stabilization and transactivation 24 h following cisplatin treatment.

The last example of nuclear phosphoinositides in the DNA damage response at longer time scales involves PI(3,4,5)P_3_-mediated selective mRNA export (Wickramasinghe et al., 2013). mRNA export from the nucleus to the cytoplasm is essential for the translation of DNA damage response proteins. mRNA export is coupled to prior steps including RNA transcription and splicing by the TREX (transcription-export) complex, which is recruited by the RNA splicing machinery. TREX components including the RNA-binding adaptor protein ALY colocalize with splicing factors in nuclear speckle domains (Masuda et al., 2005). Further studies reveal that ALY functions to release spliced mRNA from nuclear speckles for export into the cytoplasm (Dias et al., 2010). Interestingly, the mRNA export activity, as well as nuclear speckle domain residence of ALY, can be regulated *via* binding to the second messengers PI(4,5)P_2_ and PI(3,4,5)P_3_, suggesting that nuclear phosphoinositide signaling plays a role in nuclear mRNA export (Okada et al., 2008).

In this last example, Wickramasinghe et al. demonstrate that IPMK has a transcript-selective function in nuclear mRNA export in human cells, and show that its activity is necessary for the expression of certain proteins involved in HR, including RAD51, but not other related repair factors involved in NHEJ. This finding is consistent with a genetic screen in which IPMK depletion has a significant effect on HR but not NHEJ (Certo et al., 2011). In more detail, IPMK depletion or catalytic inactivation followed DNA damage by 24 h regardless of damage induction mechanism including methyl methanesulfonate, carboplatin or ionizing irradiation leads to inhibition of the nuclear export of the polyA^+^ mRNAs that encode essential HR factors such as RAD51, CHK1, or FANCD2, decreasing protein abundance. In contrast, several genes involved in NHEJ are unaffected. The downregulation of RAD51, which is a DNA repair protein essential for homologous recombination (HR) that stabilizes ssDNA at DNA lesions, provokes sensitivity to genotoxic lesions repaired by HR, and causes structural chromosome aberrations typical of defective HR. Further characterization reveals that recognition of a sequence motif in the untranslated region of RAD51 transcripts by the mRNA export factor ALY requires PI(3,4,5)P_3_, which is a product of IPMK. Exogenous PI(3,4,5)P_3_ restores ALY recognition in IPMK-depleted cell extracts, indicating a mechanism underlying transcript selection. It is therefore suggested that IPMK plays a role in a transcript-selective mRNA export pathway in response to DNA damage by regulating nuclear phosphoinositide turnover that helps to preserve genome integrity (Wickramasinghe et al., 2013).

## The Link Between Nuclear Phosphoinositide and p53 in Response to Genotoxic Stress

### Genotoxic Stress Perturbs Nuclear Phosphoinositide Homeostasis

**FIGURE 4:**
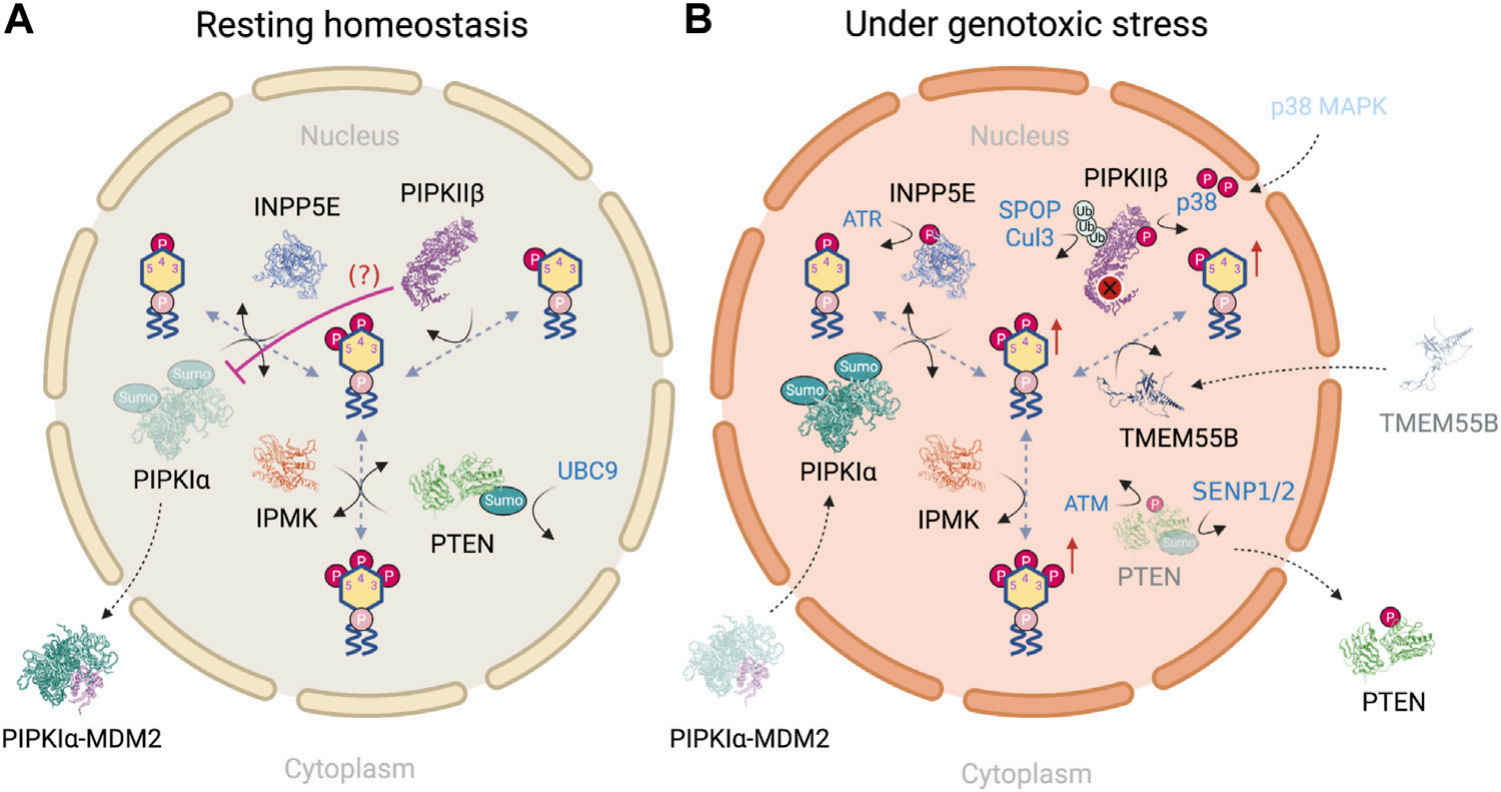
**(A)** PI(4,5)P_2_-centric view of nuclear phosphoinositide homeostasis in a resting state and **(B)** how the regulatory steps are altered in response to genotoxic stress. Figures created with BioRender.com.

In this section, we provide a PI(4,5)P_2_-centric view to show how the nuclear phosphoinositides are differentially regulated in a resting state (Figure 4A) vs under genotoxic stress (Figure 4B). We mainly focus on enzymes that have shown nuclear localization and altered activity in response to DNA damage. For example, nuclear PLCβ1 is involved in regulating nuclear PI(4,5)P_2_ fluctuations throughout the cell cycle and differentiation (Cocco et al., 2006; Ratti et al., 2017). Its dysregulation is implied in the progression from myelodisplastic syndrome to acute myeloid leukemia (Martelli et al., 2005; Fiume et al., 2010; Ratti et al., 2021). However, its regulation in response to DNA damage and/or interaction with DNA repair proteins is minimal (Piazzi et al., 2013) and is therefore neglected.

Although both PIPKIα and PIPKIIβ pathways lead to the synthesis of nuclear PI(4,5)P_2_, it is suggested that the pool of nuclear PI(4,5)P_2_ is mainly maintained by PIPKIα through the phosphorylation of PI(4)P. This conclusion is based on the following findings: first, the amount of PI(4)P is at least 20-fold higher than that of PI(5)P in nucleus (Keune et al., 2011); second [γ^32^P] phosphate labeling using isolated rat liver nuclei demonstrates that the relative labeling ratio for PI(4,5)P_2_ at the 5′- vs. 4′-OH position is approximately 1.8, suggesting that PIPKIs are the major kinases for PI(4,5)P_2_ synthesis within the nucleus (Vann et al., 1997); third, this experiment has been performed in the presence of PI4K inhibitors, wortmannin and adenosine, which inhibit PI4KIII and PI4KII, respectively (Balla and Balla, 2006). The ratio of radiolabeling of the 5′- to the 4′-OH of PI(4,5)P_2_ in the presence of wortmannin increases up to 10:1 while adenosine has no effect. These simple and elegant studies indicate that at least 90% of nuclear PI(4,5)P_2_ is derived from the PIP5K pathway (Keune et al., 2011), and indicates that PIPKIIβ regulates nuclear PI(5)P level, as will be discussed later.

As mentioned earlier, nuclear PI(4,5)P_2_ level is increased upon genotoxic stress (Wang et al., 2017; Choi et al., 2019). It’s regulation has largely been attributed to the catalytic activity of PIPKIα. Although PIPKIγi4 is another PIPKI and the only PIPKIγ subfamily member that resides in the nucleus (Schill et al., 2009), it is omitted in this picture as its regulation under DNA damage remains to be characterized. This raises the question of how PIPKIα is differentially regulated during the resting state and under genotoxic stress. Four pathways that regulate PIPKIα have been described. The first pathway is associated with the retinoblastoma protein (pRB), that is highly mutated in human tumors. pRB is a nuclear scaffold protein for lipid kinases such as PIPKIs and DAG kinases (Divecha et al., 2002; Los et al., 2007). pRB binds to PIPKI through its pocket domain, which is often mutated in human tumors, and highly activates PIPKIα in a large T antigen-regulated manner (Divecha et al., 2002). However, how pRB regulates PIPKIα upon DNA damage induction remains unclear. pRB modulates transcription of proteins that control cell cycle progression, differentiation, and is recruited to DNA damage sites in response to DNA damage (Vélez-Cruz et al., 2016). The deregulation of pRB in tumors implicates the pRB-PIPKIα complex in directing DNA damage signaling at sites of damage and/or at a transcriptional level. In the second pathway, DNA damage also triggers the nuclear translocation of protein kinase C delta (PKCδ), which is required for the initiation of apoptosis (Devries et al., 2002). PKCδ directly interacts with PIPKIα in the nucleus. PI(4,5)P_2_, presumably produced locally by PIPKIα, stimulates PKCδ kinase activity toward Star-PAP (Li et al., 2012). As a result, PKCδ does not directly regulate PIPKIα but serves as a downstream effector of PIPKIα through nuclear PI(4,5)P_2_ in regulating protein expression. Third, DNA damage-induced by etoposide for 24 h promotes polySUMOylation of PIPKIα at K33, K244 and K490 (Chakrabarti et al., 2015) as well as the nuclear translocation of PIPKIα (Chakrabarti et al., 2013). Similar nuclear translocation of PIPKIα in response to genotoxic stress has been shown with cisplatin treatment (Choi et al., 2019). Lastly, it has been suggested PIPKIα complexes with MDM2 in the cytosol in a resting state. Such an interaction is disrupted upon damage induction, which is accompanied by the nuclear translocation of PIPKIα (Choi et al., 2019). In contrast, the association between PIPKIα and wildtype p53 is not detected in unstressed cells, but is dramatically increased by DNA-damaging agents (cisplatin and etoposide) and oxidative stress (tBHQ) (Choi et al., 2019). Together, these findings indicate that nuclear PI(4,5)P_2_ level is mainly regulated by two mechanisms: one is the nuclear level of PIPKIα, that is governed by the post-translational modification of PIPKIα; the other factor is the availability of downstream PIP2 effectors in close proximity.

Recently, an additional mechanism that regulates PIPKIα activity has been reported. The loss of PIP4Ks, which includes PIPKIIα, β, γ, *in vitro* results in an unexpected increase in PI(4,5)P_2_ and a concomitant increase in insulin-stimulated production of PI(3,4,5)P_3_. A rescue using either wild-type or kinase-dead mutants of the PIP4Ks restores cellular PI(4,5)P_2_ levels and insulin stimulation of the PI3K pathway. It therefore suggests a catalytic-independent role of PIP4Ks in regulating PI(4,5)P_2_ levels (Wang et al., 2019). These effects are explained by an increase in PIP5K activity upon the deletion of PIP4Ks, which normally suppress PIP5K activity through a direct binding mediated by the N-terminal motif of PIP4K. Therefore, PIP4Ks act as negative regulators of PIP5Ks. Although this finding is based on studies in a different context than the DNA damage response and is not limited to phosphoinositides in the nucleus, the regulation mechanism might be related. On the other hand, although type I PI(4,5)P_2_ 5-phosphatase, INPP5E, potentially plays a role in maintaining genome stability (Sierra Potchanant et al., 2017) and is an enzymatic substrate of ATR upon ATR activation (Matsuoka et al., 2007), it is not clear how ATR-mediated phosphorylation affects INPP5E activity and therefore nuclear PI(4,5)P_2_ level.

The last key lipid species is PI(3,4,5)P_3_, which has been shown to be upregulated in response to DNA damage. Nuclear PI(3,4,5)P_3_ is regulated by both IPMK and class I PI3Kβ (p110β) and potentially PTEN. IPMK demonstrates greater substrate specificity than class I PI3Ks since IPMK phosphorylates only PI(4,5)P_2_ (Resnick et al., 2005). The phosphorylation of IPMK at Ser284 by protein kinase CK2 decreases the nuclear localization of IPMK (Meyer et al., 2012). Interestingly, CK2 accumulates at DNA damage sites and is involved in a wide spectrum of DNA repair mechanisms, such as NHEJ (Olsen et al., 2012), HR (Yata et al., 2012), mismatch repair (MMR) (Weßbecher et al., 2018) and nucleotide excision repair (NER) (Shah et al., 2018). It’s not clear if CK2 regulates DNA repair processes through nuclear phosphoinositide-mediated DNA damage signaling. IPMK is almost exclusively nuclear and is unaffected by wortmannin or other PI3K selective inhibitors *in vitro* (Resnick et al., 2005). The same group has shown that inhibition of p110 PI3-kinases by wortmannin prevents IPMK phosphorylation and activation (Maag et al., 2011). Although IPMK and classical PI3K phosphorylate PI(4,5)P_2_ *in vitro* in lipid mixtures or in membranes (Resnick et al., 2005), the NR5A1-PI(4,5)P_2_ complex is a substrate of IPMK, but not classical PI3K(102). By contrast, while PTEN is strongly involved in the DNA damage response, its lipid phosphatase-dependent role in this process remains elusive. PTEN is maintained in a resting state after the SUMOylation of PTEN at Lys254 by Ubc9, which drives its nuclear localization. Further, ATM-mediated phosphorylation of PTEN at Thr398 promotes the deSUMOylation by SENP1/2 and therefore the nuclear export of PTEN as part of the DNA damage response (Bassi et al., 2013). It potentially explains the mechanism by which nuclear PI(3,4,5)P_3_ is upregulated in response to DNA damage, assuming the kinase activity of IPMK is not significantly altered during this process.

### Roles of Different Lipid Species and Their Interaction With p53

In the previous section, genotoxic stress leads to increased levels of PI(5)P, PI(4,5)P_2_ and PI(3,4,5)P_3_ in the nucleus. Accumulating evidence suggests that these changes affect p53 activities, which include a variety of responses following DNA damage, such as cell cycle arrest, DNA repair, senescence and apoptosis. Here we summarize related findings and discuss how nuclear phosphoinositide signaling impacts regulation of p53 function.

**FIGURE 5:**
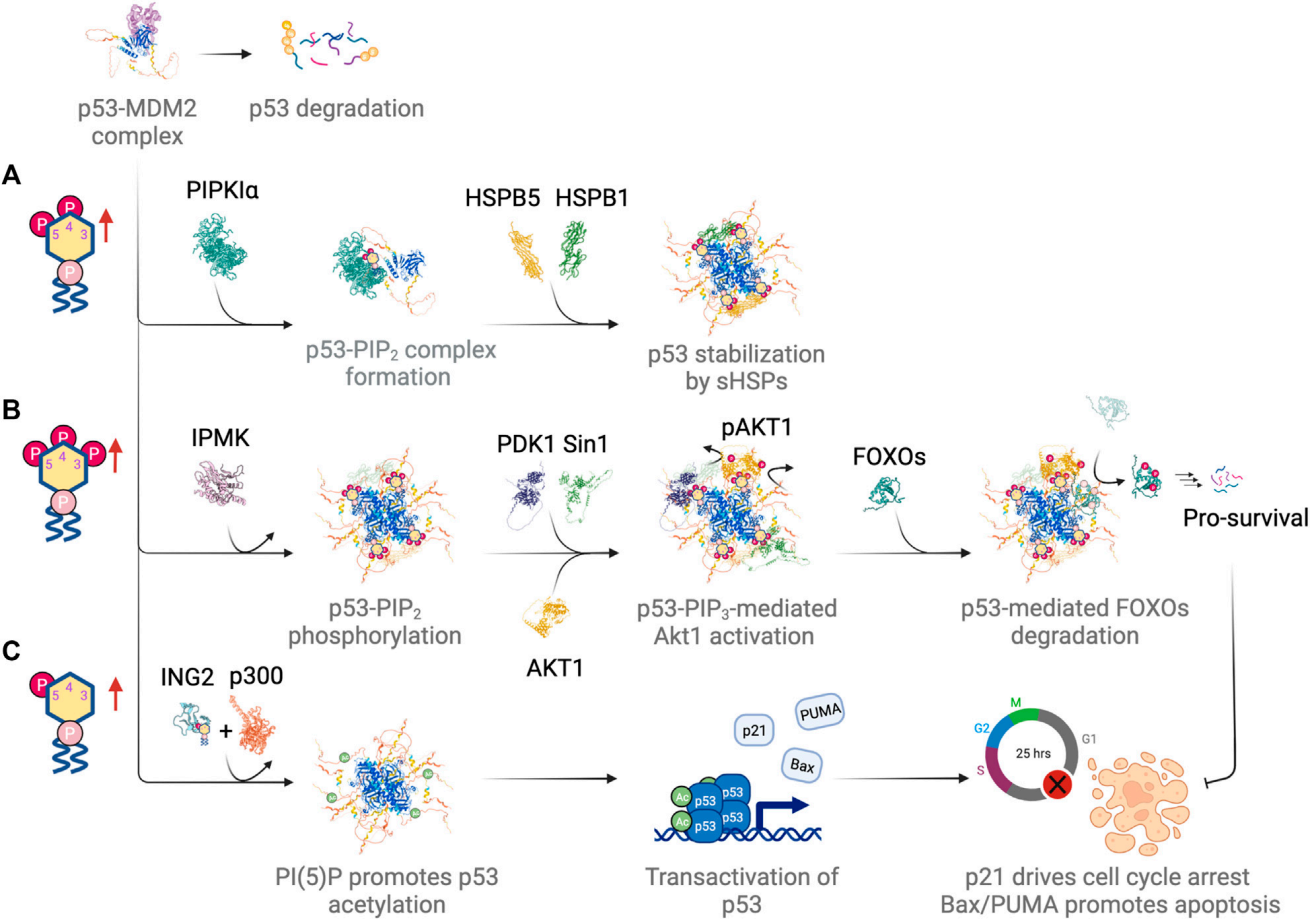
Differential regulation of p53 by different nuclear phosphoinositide species in response to DNA damage. **(A)** PI(4,5)P_2_ drives complex formation with p53 and promotes p53 stabilization by recruiting small heat shock proteins (sHSPs). **(B)** IPMK phosphorylates p53-PI(4,5)P_2_ complex, which serves as a signaling hub for Akt activation and subsequent phosphorylation and degradation of FOXOs. **(C)** PI(5)P promotes chromatin association of ING2. It drives p300-mediated acetylation and transactivation of p53. Figures created with BioRender.com.

Our previous studies showed that increased PI(4,5)P_2_ speckles induced by UVC damage strongly co-localized with γH2AX foci (Wang et al., 2017). The upregulation of nuclear PI(4,5)P_2_ due to cisplatin-induced genotoxic stress was also observed in a different study and was linked to a complex formation with p53 (Choi et al., 2019). The close contact between PIPKIα, PI(4,5)P_2_ and p53 (within 40 nm apart) has been confirmed using a proximity ligation assay and all were found to strongly co-localize with γH2AX foci. The association between PIPKIα and MDM2 was disrupted upon cisplatin treatment and it was accompanied by enhanced complex formation between PIPKIα and wildtype p53 in the nucleus, which was otherwise undetected in a resting state. Interestingly, PIPKIα formed stable complexes with mutant p53s even in a resting state. In the case of mutant p53s, PIPKIα binding was required for the stabilization of mutant p53s at a high expression level. PIPKIα interacted with p53 through the p53 oligomerization domain (OD) and CTD. The production of PI(4,5)P_2_ in the close vicinity of p53 promoted the association of Hsp27 and/or HspB1 but attenuated the association of PIPKIα with p53 (Figure 5A). Both PIPKIα depletion and PIPKIα inhibitor, ISA-2011B, impaired the association of Hsp27 with p53. By contrast, mutant p53 stabilization depends on its constant complex formation with PIPKIα and Hsp70 or Hsp90 chaperone proteins. Hsp70 and Hsp90 were postulated to block access of E3 ligase to mutant p53. However, Hsp90 is mostly cytosolic and Hsp70-p53 binding does not require p53-PI(4,5)P_2_ for complex formation. Additionally, the binding of PI(4,5)P_2_ prevents p53 from interacting with MDM2, which is another mechanism to stabilize p53. The binding of PI(4,5)P_2_ to p53 is mapped to the C-terminal polybasic stretch of p53, which indicates electrostatic interactions play a major role when interacting with phosphoinositide lipids in nucleus (Mazloumi Gavgani et al., 2021). Surprisingly, the PI(4,5)P_2_-p53 interaction was very stable and was resistant to denaturation during SDS-polyacrylamide gel electrophoresis (SDS-PAGE) (Choi et al., 2019). Altogether, nuclear PI(4,5)P_2_ mediated p53 stabilization offers a mechanism different from the canonical Hsp70-Hsp90-MDM2 pathway and its dysregulation has direct implications in tumorigenicity.

Multiple roles have been suggested for PI(3,4,5)P_3_-mediated regulation of p53. In one case, the p53-PI(3,4,5)P_3_ complex served as a signaling hub for Akt signaling (Chen et al., 2021a). Activation of the nuclear Akt pathway involved the kinase activity of IPMK, which turned the p53-PI(4,5)P_2_ complex into p53-PI(3,4,5)P_3_. The complex then recruited Akt as well as the PI(3,4,5)P_3_-binding kinases PDK1 and Sin1. The latter was a subunit of the mTORC2 complex. Both PDK1-dependent phosphorylation of Akt at Thr308 and Sin1-dependent phosphorylation of Akt at Ser473 activated the nuclear Akt pathway, which has been shown to be required for p53 stabilization (Boehme et al., 2008). This led to the phosphorylation of the forkhead box O (FOXO) protein family, which included FOXO1, FOXO3, FOXO4 and FOXO6. p53-mediated phosphorylation of FOXO proteins by Akt promoted the degradation of FOXOs, which was a pro-survival signal and inhibited DNA damage-induced apoptosis (Figure 5B). FOXOs directly interacted with p53 (Wang et al., 2008) and p53 promoted FOXOs degradation through MDM2-mediated ubiquitination (Fu et al., 2009).

Surprisingly, other than mediating p53-dependent Akt activation, IPMK also functions as a transcriptional coactivator of p53 (Xu et al., 2013) (reviewed elsewhere (Kim et al., 2017)). IPMK overexpression increases transcriptional activity of p53 as reflected in mRNA levels for canonical p53 targets (e.g., PUMA, Bax, and p21) (Xu et al., 2013; Xu and Snyder, 2013). The increase in p53 transcriptional activity following etoposide treatment also depends on IPMK. IPMK binds to p53 directly and stimulates its acetylation by binding to p300, an acetyltransferase. IPMK depletion disrupts the p53-p300 interaction, which leads to a decreased acetylation of both p53 and histones, and thereby reduces the transcription of p53 target genes and p53-mediated apoptosis. Interestingly, IPMK-dependent p53 acetylation is independent of IPMK kinase activity, since overexpression of the kinase deficient mutant of IPMK (K129A-S235A) in U2OS causes a similar increase in p53-IPMK interaction upon etoposide treatment as with wildtype IPMK.

PTEN also contributes to p53 regulation in both a phosphatase-dependent and phosphatase-independent manner (Freeman et al., 2003; Trotman and Pandolfi, 2003). While p53 is known to drive PTEN expression following DNA damage such as γ irradiation (Stambolic et al., 2001), the effect of PTEN on p53-mediated transcription is also known. PTEN, but not its phosphatase-dead mutant, is able to produce a moderate increase in p53-mediated transcription in the presence of MDM2, indicating that PTEN can indirectly protect p53 from MDM2-mediated degradation (Mayo et al., 2002). Intriguingly, a molecular interaction between PTEN and p53 is mapped to the C-terminal of p53, where PI(4,5)P_2_ binds (Choi et al., 2019), using GST-pulldown of p53 partial deletion mutants (Freeman et al., 2003). The association of p53, on the other hand, is mapped to the C2 domain of PTEN, which is a Ca^2+^-dependent membrane-targeting module (Lee et al., 1999; Georgescu et al., 2000; Yasui et al., 2014). However, phosphatase-dead mutants of PTEN can also protect p53 from MDM2-independent degradation. Addition of a phosphatase-dead PTEN mutant or the PTEN C2 domain also results in the elevation of p53 level, consistent with a role for PTEN in p53 stabilization through direct interaction (Freeman et al., 2003). PTEN also complexes with p300 in the nucleus and plays a role in maintenance of high p53 acetylation in response to DNA damage (Li et al., 2006). Intriguingly, p53 acetylation not only promotes PTEN-p53 interaction but also promotes p53 tetramerization. Importantly, PTEN is unable to induce the transactivation of the acetylation-deficient mutant of p53 (p53-5 KR), which has all five acetylation sites, Lys370, 372, 373, 381, and 382, mutated. This indicates that p53 acetylation is required for activation of p53 by PTEN and subsequent maintenance of high p53 acetylation (Li et al., 2006). Interestingly, these sites are essentially the same sites for p53-PI(4,5)P_2_ interaction (Choi et al., 2019).

Finally, we consider the consequences of nuclear PI(5)P upregulation in p53 function (Figure 5C). As mentioned previously, the upregulation of nuclear PI(5)P leads to the binding of ING2 (inhibitor of growth family member 2) to PI(5)P and thereby promotes ING2 association with chromatin upon DNA damage (Gozani et al., 2003; Zou et al., 2007). ING2 is a component of a chromatin-regulatory complex that represses a specific subset of genes in response to DNA damage (Bua et al., 2013). ING2-mediated gene repression is through the interaction between ING2 and H3K4me3, which stabilizes ING2-SIN3A-HDAC1 complexes at target gene promoters and promotes histone deacetylation (Shi et al., 2006; Smith et al., 2010). ING2 contains a PHD finger domain that coordinates with zinc and binds to PI(5)P (Gozani et al., 2003). Possibly, the generation of PI(5)P at specific target sites on chromatin recruits and activates ING2 (Gozani et al., 2003). The loss of ING2 binding to PI(5)P reduces p53-mediated apoptosis in response to etoposide or H_2_O_2_ (Gozani et al., 2003). This is consistent with studies that show ING2 upregulation and increases in nuclear localization of Type I PI(4,5)P_2_ 4-phosphatase, TMEM55B, the main PI(5)P generating enzyme after DNA damage (Nagashima et al., 2001; Zou et al., 2007; Bua et al., 2013). Interestingly, similar to IPMK, ING2 also complexes with the histone acetyltransferase p300. It enhances the interaction between p53 and p300 as well as being a cofactor for p300-mediated p53 acetylation (Nagashima et al., 2001; Pedeux et al., 2005). Furthermore, overexpression of ING2 induces senescence in young fibroblasts in a p53-dependent manner. The level of ING2 expression directly modulates the onset of replicative senescence (Pedeux et al., 2005). Taken together, these studies show that PI(5)P plays a key role in localizing ING2 to chromatin and activating it in response to genotoxic and oxidative stress.

## Nuclear Phosphoinositides in Genome Stability and Tumor Suppression

### A p53-Centric View of DNA Damage Repair Pathway Selection

Next, we focus on new findings that provide a different view of p53’s role in DNA damage repair, which involves repair pathway choice. In a commonly accepted model, the downstream transactivation of p53 is considered its major role in response to DNA damage. p53 helps maintain genome stability by promoting the expression of proteins involved in cell cycle arrest, DNA repair, apoptosis and senescence. However, the roles of p53 as a tumor suppressor cannot be fully explained by the transcriptional activity of p53. In this last section, we provide a p53-centric view and focus on an upstream and transcription-independent function of p53 in directing DNA repair pathway selection that strongly correlates with its tumor suppressive activity.

p53 was known to direct repair pathway choice (Sengupta and Harris, 2005). This feature was highlighted in recent years due to advances in genomic editing using CRISPR-Cas9. Two independent studies both pointed out that wildtype p53 inhibited Cas9-mediated genomic editing by suppressing homologous recombination (Haapaniemi et al., 2018; Ihry et al., 2018). By contrast, p53 depletion increased the rate of homologous recombination and inhibited the DNA damage response. This finding led to a dilemma when creating genomic-edited cells for therapeutic purposes because successfully edited cells might have inherited defects in p53 function and were therefore more likely to be tumorigenic.

**FIGURE 6:**
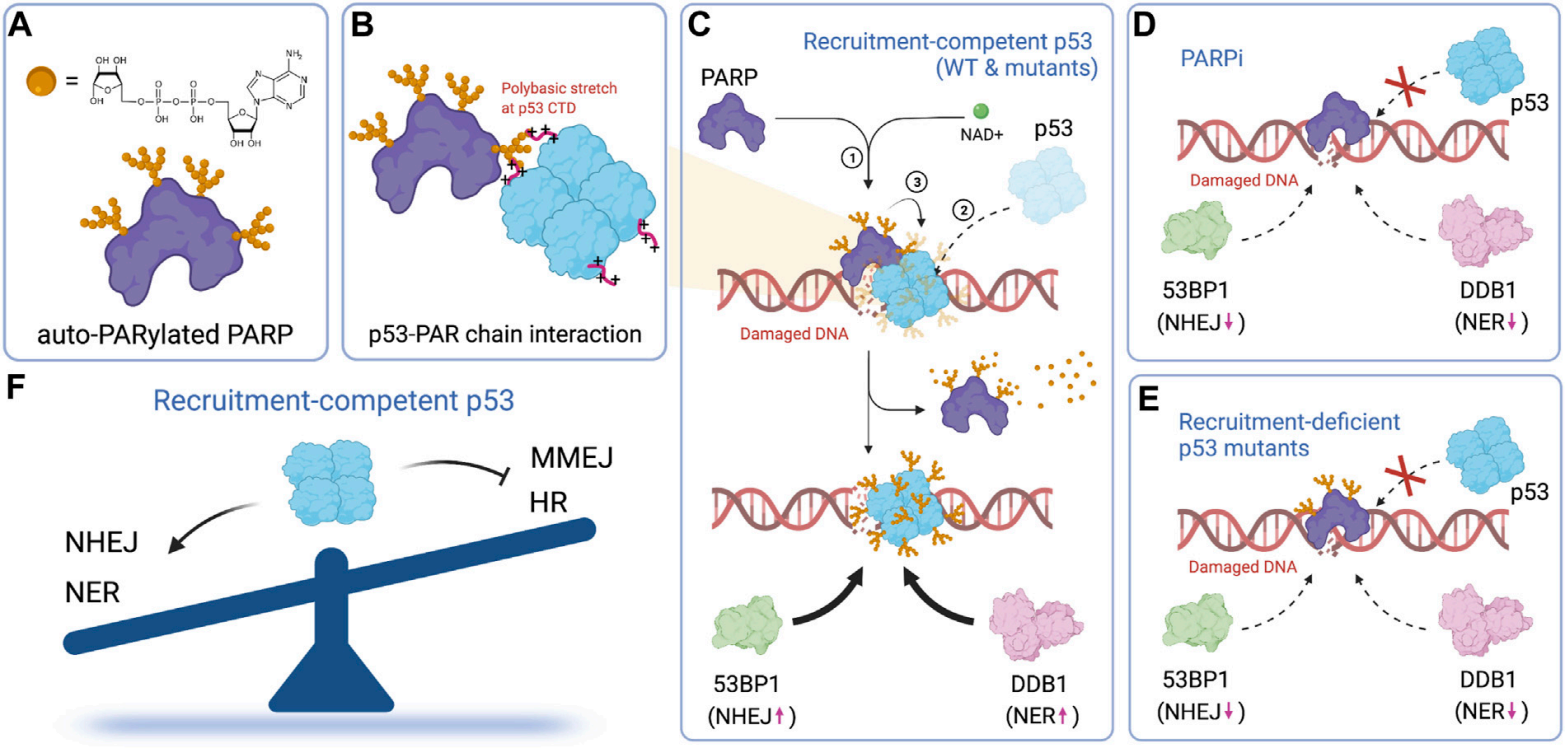
PARP-dependent rapid recruitment of p53 directs repair pathway choice. **(A)** Chemical structure of ADP-ribose and the illustration of auto-PARylated PARP. **(B)** p53 interacts with auto-PARylated PARP through its C-terminal polybasic stretch. **(C)** Auto-PARylated PARP drives the accumulation of p53, which promotes the recruitment of 53BP1 and DDB1 and therefore NHEJ and NER, respectively. **(D)** The recruitment of p53, as well as the subsequent recruitment of 53BP1 and DDB1, is suppressed by a PARP inhibitor. **(E)** Mutations that impair p53 rapid recruitment also suppress recruitment of 53BP1 and DDB1. **(F)** Early recruitment of p53 tips the balance of repair pathway selection in favor of the NHEJ and NER repair pathway in a transcription-independent fashion. Figures created with BioRender.com.

Repair pathway selection is an early event in the DNA damage response. It likely relies on transcription-independent functions, rather than transactivation of p53. Several studies focus on the transcription-independent functions of p53 in DNA repair (Williams and Schumacher, 2016; Ho et al., 2019). One proposed mechanism by which p53 suppresses homologous recombination (HR) is through p53-mediated sequestering of Rad51 (Linke et al., 2003). In this model, direct binding of p53 to Rad51 through its C-terminal domain prevents Rad51 polymerization on ssDNA and hence HR. Our recent work provides an alternative mechanism (Wang et al., 2022). We show that PARP-dependent modification of p53 enables rapid recruitment of p53 to damage sites within a few seconds of laser microirradiation-induced DNA damage (Figures 6A–D). Rapid recruitment of p53 requires both its DNA-binding domain (DBD) and C-terminal domain (CTD) and is uncorrelated with its transcriptional activity. The rapid recruitment of p53 promotes the subsequent recruitment of upstream repair factors including 53BP1, whose recruitment promotes the NHEJ pathway (Escribano ‐ Diaz and Durocher, 2013; Gupta et al., 2014; Swift et al., 2021), and DDB1, whose recruitment initiates nucleotide excision repair (NER) (Figures 6C,E). The clearance efficiency of UV-specific damage by the NER pathway strongly depends on the early recruitment of p53. This is evident when comparing the repair efficiencies of recruitment-competent (P47S, R175P and the double QS mutant) vs recruitment-deficient (R175H, R278Q, ΔCTD) mutants of p53 (Figure 6F). Our model helps explain how tumor-derived p53 mutants promote, rather than suppress HR (Bertrand et al., 1997; Saintigny et al., 1999; Saintigny and Lopez, 2002; Linke et al., 2003), although the CTD of tumor-derived p53 mutants remain intact and are capable of interacting with Rad51. It also provides an explanation regarding how certain transcriptionally-deficient p53 mutants retain tumor suppressive functions.

### Roles of Nuclear Phosphoinositide in p53-Mediated Tumor Suppression

Because p53 mutation is involved in almost 50% of cancers, nuclear phosphoinositide-mediated regulation of p53 potentially provides a novel entry for developing therapeutic strategies. Moreover, p53 governs not only tumor suppression, but also many aspects of cellular functions including DNA repair (Sengupta and Harris, 2005; Williams and Schumacher, 2016), cell cycle arrest (Chen, 2016) and apoptosis (Aubrey et al., 2018) etc. This topic is too broad to be covered in one section and many of these topics have been separately reviewed elsewhere. Here we would like to highlight three aspects of nuclear phosphoinositide-mediated p53 regulation in human diseases.

The first is the role of PIP4Ks in tumor suppression. The question of the roles of PIP4Ks in p53-null tumors has been addressed in mouse xenograft models (Emerling et al., 2013). Those studies show that PIP4K depletion reduces tumor-dependent death in p53 null mice. At a cellular level, knockdown of PIPKIIα and PIPKIIβ increases metabolic stress and ROS, leading to senescence in a cell line lacking p53 (BT474) but not in MEFs in the same experiment. This observation indicates that partial loss of *PIP4K2A* and/or *PIP4K2B* alleles (genes encoding PIPKIIα and PIPKIIβ, respectively) may result in senescence in the context of p53-deficient tumors. Knockout of either *PIP4K2B* or *PIP4K2A* in a wildtype p53 background did not lead to a strong phenotype in both mice and tissue culture studies (Wang et al., 2019). By contrast, no mice with the *PIP4K2B* ^−/-^, *TP53* ^−/−^ genotype emerged from the crosses, indicating synthetic lethality (Lamia et al., 2004). Additionally, *PIP4K2B* ^−/-^ mice showed increased sensitivity to insulin stimulated Akt activation in muscle compared to their wildtype counterparts (Lamia et al., 2004). Mice embryos with double knockout of both *PIP4K2A* and *2B* developed normally, but died within 12 h after birth (Emerling et al., 2013). Mice with germline deletion of *PIP4K2C* also appeared normal in regard to growth and viability but had increased inflammation and T-cell activation as they aged (Shim et al., 2016). Since the double KO of *PIP4K2A* and *2B* is prenatal lethal for the mice, the Cantley laboratory crossed *PIP4K2A* ^−/-^, *PIP4K2B* ^+/−^ mice with *TP53* ^−/−^ mice to investigate the effect of partial loss of PIP4Ks in a p53-null background and got viable litters. While p53-deficient mice developed spontaneous tumors in approximately 4–6 months (Jacks et al., 1994), the litters from the crosses had a dramatic reduction in tumors compared to the *PIP4K2A* ^+/+^, *PIP4K2B* ^+/+^, *TP53* ^−/−^ mice. These findings stimulated the recent development of PIP4K inhibitors, which included covalent (Manz et al., 2020; Sivakumaren et al., 2020) and non-covalent inhibitors (Chen et al., 2021b). Excitingly, the noncovalent and selective inhibitors also demonstrated selective killing of p53-null tumor cells with disruption of cell energy metabolism (Chen et al., 2021b) (see review (Fiume et al., 2015)). Together, these results indicated that expression of PIPKIIα and/or β was critical for the growth of tumors with *TP53* mutations or deletions, which made PIP4Ks a promising druggable target for cancer treatment.

The second aspect is the complex formation between mutant p53, nuclear PIPKIα and PI(4,5)P_2_, which has direct implications in tumorigenesis (Choi et al., 2019). Recent studies from the Anderson laboratory showed that depletion of PIPKIα or inhibition of its activity using a PIPKI-α inhibitor, ISA-2011B (Semenas et al., 2014), decreased the level of mutant p53. Treatment with ISA-2011B also led to a reduction of PIPKIα. By contrast, PIPKIα knockdown or ISA-2011B had no effect on wildtype p53 protein levels under unstressed conditions. While the association between wildtype p53 and PI(4,5)P_2_ was promoted by DNA damage, cancer cells bearing p53 mutations showed a strong interaction between p53 and PI(4,5)P_2_ even in resting states. The p53-PI(4,5)P_2_ interaction has been mapped to the CTD of p53 but it did not provide an explanation regarding how tumor-derived p53 mutants, which often carry mutations in the DBD, were differentially regulated by PI(4,5)P_2_ that interacted with p53 through the CTD of p53. Deciphering the molecular functions of such stable interactions between PI(4,5)P_2_ and mutant p53 is important.

The differential regulation of wildtype vs mutant p53 by a nuclear PI(4,5)P_2_ pathway has profound implications for cancer. The stability of wildtype and mutant p53s has been reported to be regulated by distinct mechanisms (Freed-Pastor and Prives, 2012). The stable association of PI(4,5)P_2_ with mutant p53 is unexpected and may be an underlying mechanism for the long-term stability of mutant p53s. Although PIPKIα, mutant p53 and sHSPs are independently implicated in tumor progression (Arrigo and Gibert, 2014; Muller et al., 2014; Semenas et al., 2014; Malin et al., 2016), findings indicate that these proteins form an orchestrated molecular complex that may play a central role in tumorigenesis. In addition, αB-crystallin expression correlates with p53 protein stabilization in clinically aggressive triple-negative breast cancers (Koletsa et al., 2014) and the gene encoding PIPKIα is commonly amplified in breast cancer (Waugh, 2014), indicating that this complex may contribute to the pathogenesis of these and perhaps other cancers. Given the critical role of mutant p53 stability in its oncogenic activity (Freed-Pastor and Prives, 2012; Muller et al., 2014; Alexandrova et al., 2015), these findings indicate that the PIPKIα and PIPKIα–p53–PI(4,5)P–sHSP complexes might serve as therapeutic targets for cancer.

Finally, there is a potential role for nuclear phosphoinositides in synthetic lethality by p53-mediated DNA damage repair pathway selection. As discussed previously, we find that the rapid recruitment of p53 is a newly identified property that is independent of its transcriptional activity. The ability of p53 or its mutants to be rapidly recruited to damage sites correlates with their tumor suppressive function *in vivo*. The finding that p53 interacts with PI(4,5)P_2_ upon DNA damage brings in additional factors in this process. Because the rapid recruitment of p53 strongly relies on its CTD, it appears that the polybasic stretch of p53 is critical for its interaction with auto-PARylated PARP and subsequent p53 PARylation (Wang et al., 2017; Fischbach et al., 2018). Interestingly, the same polybasic stretch is critical for p53’s interaction with PI(4,5)P_2_ (Choi et al., 2019). This implies that PI(4,5)P_2_ might play a role in regulating the PARylation of p53, which is required for its rapid recruitment to DNA damage sites and hence repair pathway selection. Furthermore, a recent interactome study validates PARP1 as a PI(4,5)P_2_-binding protein (Mazloumi Gavgani et al., 2021). The interactions between PARP1, PI(4,5)P_2_/PI(3,4,5)P_3_ and p53 form a new signaling axis and how nuclear PI(4,5)P_2_ affects DNA repair pathway selection warrants further investigation.

## Conclusion

Nuclear phosphoinositides play a role in maintaining genomic stability. Genomic stability is under constant threat from both endogenous and exogenous factors, and its disruption is a hallmark of cancer (Negrini et al., 2010; Hanahan, 2022). Replication fidelity and repair of damaged DNA ensures correct genetic information is transmitted during cell division and proliferation. These processes are critical to genomic integrity and even slight deviations can result in age-associated diseases and cancer (Hoeijmakers, 2001). Further, emerging evidence shows that many of these processes involve nuclear phosphoinositides. In this review, we have focused on the roles of nuclear phosphoinositides in regulating the DNA damage response. Our recent evidence indicates that there is a basic mechanism of DNA repair pathway selection by p53 likely involving PI(4,5)P_2_. The signaling of nuclear phosphoinositides in response to DNA damage and particularly their roles in p53-dependent repair pathway selection potentially provide new handles for treatments through synthetic lethality.
